# Potential Significance of Targeting Ferroptosis for Intervention of Diabetic Cardiomyopathy

**DOI:** 10.1111/1753-0407.70116

**Published:** 2025-06-19

**Authors:** Qian Lei, Burton B. Yang, Juanjuan Lyu

**Affiliations:** ^1^ Anesthesia and Operation Center, Sichuan Academy of Medical Sciences and Sichuan Provincial People's Hospital University of Electronic Science and Technology of China Chengdu China; ^2^ School of Medicine University of Electronic Science and Technology of China Chengdu China; ^3^ Sunnybrook Research Institute, and Department of Laboratory Medicine and Pathobiology University of Toronto Toronto Canada; ^4^ Department of Pediatrics, West China Second University Hospital Sichuan University Chengdu China; ^5^ Key Laboratory of Birth Defects and Related Diseases of Women and Children, Ministry of Education, West China Second University Hospital Sichuan University Chengdu China

**Keywords:** diabetes, diabetic cardiomyopathy, ferroptosis, lipid peroxidation, reactive oxygen species

## Abstract

**Background:**

Diabetes represents a significant global health concern, with diabetic cardiomyopathy (DCM) emerging as a primary cause of mortality among individuals with diabetes. Despite the prevalence of DCM, advancements in therapeutic and preventative strategies remain constrained.

**Methods:**

Recent studies were reviewed to provide a comprehensive summary of pathogenesis and clinical treatment of DCM, effect of ferroptosis, and potential value of ferroptosis inhibitors in DCM intervention.

**Results:**

A growing body of research indicates that oxidative stress, inflammatory reactions, and other factors play a role in the onset and progression of DCM. Oxidative stress within cardiomyocytes is a primary mechanism implicated in the development of DCM, whereby heightened intracellular reactive oxygen species (ROS) facilitate cell death via ferroptosis. Ferroptosis inhibitors hold great promise as therapeutic agents.

**Conclusions:**

This review provides an overview of the involvement of iron homeostasis regulation, oxidative stress, and ferroptosis in DCM, and the significance of ferroptosis in the prevention and treatment of DCM.

## Graphical abstract

The pathophysiology of DCM encompasses insulin resistance and hyperglycemia‐induced metabolic alterations in cardiomyocytes, mitochondrial dysfunction and oxidative stress, cardiac lipotoxicity, dysregulation of immune responses, endoplasmic reticulum (ER) stress, impaired calcium handling, and activation of the renin‐angiotensin‐aldosterone system (RAAS). Oxidative stress results in cardiomyocyte hypertrophy, myocardial fibrosis, cardiomyocyte ferroptosis and immune property modulation and ultimately lead to HFpEF. Ferroptosis inhibitors hold great promise as therapeutic agents for the intervention of DCM.

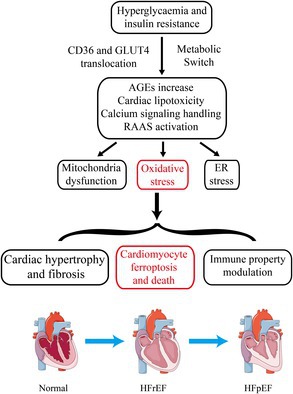

**Abbreviations**
AGEs: advanced glycation end products
CoQ10: coenzyme Q10
DCM: diabetic cardiomyopathy
FSP1: ferroptosis suppressor protein 1
GLP‐1: glucagon‐like peptide‐1
GLUT4: glucose transporter 4
H/R: hypoxia‐reoxygenation
HF: heart failure
LVEF: left ventricular ejection fraction
NF‐κB: nuclear factor‐κB
PPARα: peroxisome proliferator‐activated receptor α
RAAS: renin‐angiotensin‐aldosterone system
ROS: reactive oxygen species
SGLT: sodium‐glucose cotransporter
SGLT‐2: sodium‐glucose cotransporter‐2
SLC7A11: solute carrier family 7 member 11
STZ: streptozotocin
T1DM: type 1 diabetes mellitus

**Summary**

- Diabetic cardiomyopathy (DCM) is a serious complication of diabetes mellitus, characterized by structural and functional abnormalities of the heart muscle.
- Ferroptosis has been shown to play a pivotal role in the development and progression of DCM.
- Several ferroptosis inhibitors hold great promise as therapeutic agents for the intervention of DCM.
- This review provides an overview of the involvement of ferroptosis in DCM, and the significance of ferroptosis in the prevention and treatment of DCM was systematically discussed.

Diabetes poses a significant medical challenge globally [1, 2], with cardiovascular disease being the primary cause of mortality among diabetic individuals [3]. Diabetic cardiomyopathy (DCM), a specific form of heart disease linked to diabetes, is characterized by cardiac dysfunction in the absence of traditional risk factors such as coronary artery disease or hypertension [3]. Despite being proposed by Shirley Rubler in 1972 [4], the clinical prognosis of DCM remains unfavorable. The global incidence of diabetes in 2021 was 529 million individuals, a number projected to increase to 1.31 billion by 2050 [1]. Of these cases, over 90% are attributed to T2DM mellitus (T2DM) [1]. Cardiovascular disease is the primary cause of mortality among diabetic patients, regardless of gender. Diabetes is a significant risk factor for heart dysfunction and heart failure (HF), independent of age and other comorbidities. The prevalence of HF in individuals with T2DM is significantly elevated compared to those without diabetes, with men experiencing a 2.4‐fold increase and women a 5.1‐fold increase in risk [5, 6, 7]. Diabetes contributes to a substantial portion of cases of clinical HF and is a significant predictor of unfavorable outcomes [8, 9]. Despite the high incidence of DCM, advancements in therapeutic and preventative strategies for this condition remain limited. Effective management of DCM necessitates a careful balance between glycemic control and cardiovascular protection. Several anti‐HF medications demonstrate comparable efficacy in treating patients with diabetes and those without diabetes. Nevertheless, conventional hypoglycemic agents may offer limited protection for the cardiovascular system [10]. Novel glucose‐controlling drugs glucagon‐like peptide 1 (GLP‐1) receptor agonists and sodium‐glucose cotransporter 2 (SGLT2) inhibitors have positive performance in reducing the burden on the heart and are positive changes in the control of DCM [11]. However, there are no tailored therapeutic interventions specifically formulated for DCM.

Increasing evidence suggests that oxidative stress and inflammatory processes play significant roles in the pathogenesis of DCM [3]. Oxidative stress within cardiomyocytes represents a primary mechanism underlying the development of DCM. Consequently, heightened intracellular levels of reactive oxygen species (ROS) facilitate cellular demise via ferroptosis, a distinctive mode of cell death distinguished by the buildup of iron‐mediated lipid peroxidation. This paper provides an overview of the regulatory mechanisms governing iron homeostasis, oxidative stress, and ferroptosis in DCM, and comprehensively examines the importance of ferroptosis in the management and therapy of DCM, integrating insights from clinical and preclinical drug trials in the field.

## 1 The Pathogenesis and Clinical Treatment of DCM

### 1.1 The Pathogenesis of DCM

DCM typically manifests as HF resulting from impaired left ventricular function. HF is a chronic and progressive syndrome caused by structural or functional abnormalities in the heart [12]. Classification of HF is based on the left ventricular ejection fraction (LVEF), with HF categorized as reduced ejection fraction (HFrEF) when LVEF is ≤ 40%, mildly reduced ejection fraction (HFmrEF) when LVEF is between 41% and 49%, and preserved ejection fraction (HFpEF) when LVEF is ≥ 50% [13]. HF with HFrEF is precipitated by the impairment of systolic function, resulting in myocardial stretching and subsequent thinning and weakening of the heart muscle. Conversely, HF with HFpEF is characterized by a preserved LVEF and is attributed to the impairment of diastolic function. HFpEF is distinguished by ventricular stiffening, thickening, and cardiac enlargement [14].

The onset and progression of DCM are influenced by various contributing factors. The pathophysiology of DCM encompasses a complex interplay of molecular mechanisms, including insulin resistance and hyperglycemia‐induced metabolic alterations in cardiomyocytes, mitochondrial dysfunction and oxidative stress, cardiac lipotoxicity, dysregulation of immune responses, endoplasmic reticulum (ER) stress, impaired calcium handling, and activation of the renin‐angiotensin‐aldosterone system (RAAS). These molecular alterations ultimately result in cardiomyocyte hypertrophy, myocardial fibrosis, and microvascular dysfunction, culminating in the development of cardiac stiffness, diastolic dysfunction, and HF [15].

#### 1.1.1 Cardiomyocyte Metabolic Switch

In healthy status, the energy metabolism of cardiomyocytes primarily relies on fatty acids [16]. However, in individuals with type 1 diabetes mellitus (T1DM) and T2DM, there is an observed increase in fatty acid uptake and a decrease in glucose oxidation in both human patients and animal models of DCM [17, 18]. Insulin resistance and hyperglycemia can stimulate the peroxisome proliferator‐activated receptor α (PPARα), leading to enhanced translocation of the fatty acid translocase platelet glycoprotein 4 (CD36) to the sarcolemma [19], thereby facilitating the uptake of fatty acids. The impairment of the insulin signaling pathway can hinder the translocation of glucose transporter 4 (GLUT4) [20], resulting in alterations in CD36 and GLUT4 levels that prompt a shift in the metabolic profile of cardiomyocytes from glucose metabolism to fatty acid β‐oxidation. This transition reduces metabolic efficiency and induces metabolic stress in the failing heart [21]. Elevated intracellular fatty acid levels in cardiomyocytes contribute to cardiac lipotoxicity and hinder autophagy and insulin signaling pathways, which will be further elaborated upon.

Hyperglycemia contributes to the deterioration of cardiac structure and function by inducing the production of ROS, which inhibit the activity of glyceraldehyde 3‐phosphate dehydrogenase and promote the formation of advanced glycation end products (AGEs) [22]. AGEs, in turn, stimulate collagen expression, accumulation, and crosslinking, ultimately leading to increased myocardial fibrosis [23]. Additionally, AGEs bind to the receptor for AGEs, triggering the activation of the nuclear factor‐κB (NF‐κB) signaling pathway and the subsequent release of proinflammatory cytokines, chemokines, and exosomes from inflammatory cells [24]. Elevated glucose levels in individuals with diabetes mellitus have been shown to contribute to the development of DCM by increasing the O‐GlcNAcylation modification of various proteins, which in turn alters Ca^2+^ uptake, impairs mitochondrial function, and disrupts autophagy [20].

#### 1.1.2 Cardiac Lipotoxicity

In DCM, heightened fatty acid uptake results in the excessive activation of β‐oxidation, leading to the accumulation of incompletely metabolized fatty acids within the myocardium. This lipid deposition within myocardial cells ultimately contributes to myocardial dysfunction. Cardiac lipotoxicity can induce disturbances in energy metabolism within myocardial cells, eliciting inflammatory and oxidative stress responses that contribute to cellular damage and exacerbate myocardial dysfunction [3, 22]. Additionally, polyunsaturated fatty acids within myocardial cells may heighten susceptibility to ferroptosis.

#### 1.1.3 Activation of RAAS

The RAAS is a stress system comprised of enzymes and peptides that primarily regulate vasoconstriction, sodium reabsorption, and fluid balance [25]. In DCM, the RAAS is stimulated as a result of metabolic dysfunction, insulin resistance, and various other factors, resulting in elevated levels of angiotensin II and other bioactive molecules. These bioactive molecules have the capacity to induce hypertrophy and fibrosis in myocardial cells, consequently worsening myocardial dysfunction [26]. Additionally, RAAS plays a role in the modulation of oxidative stress, autophagy, and apoptosis pathways in myocardial cells [21]. The RAAS is a critical factor in the pathogenesis of DCM, impacting the physiological function of myocardial cells through the modulation of vasoconstriction, sodium reabsorption, and fluid homeostasis. Consequently, the management of RAAS holds considerable importance in the prevention and treatment of DCM [3].

#### 1.1.4 Mitochondrial Dysfunction and Oxidative Stress

In DCM, dysregulated metabolism, insulin resistance, and other contributing factors lead to heightened ROS production within myocardial cells, resulting in increased oxidative stress reactivity and susceptibility to autophagy and apoptosis [27, 28, 29]. The pathophysiology of oxidative stress in diabetes‐induced cardiomyopathy is a multifaceted process influenced by various factors. Oxidative stress induces myocardial cell damage, resulting in cell death and interstitial fibrosis, ultimately impacting cardiac function. ROS generated during fatty acid metabolism are implicated in myocardial cell injury. Furthermore, oxidative stress disrupts mitochondrial function in myocardial cells, leading to diminished ATP synthesis and decreased energy metabolism efficacy [15, 30, 31].

#### 1.1.5 ER Stress and Impaired Calcium Handling

ER stress, a cellular response to unfolded proteins, is implicated in the pathogenesis of DCM. Metabolic dysfunction, insulin resistance, and other factors in DCM lead to impairment of myocardial ER function, resulting in ER stress. This stress exacerbates the progression of DCM. Activation of signal transduction pathways, such as the protein kinase R‐like endoplasmic reticulum kinase and activating transcription factor 6 pathways, occurs in response to ER stress in myocardial cells. The activation of signaling molecules initiates a cascade of cellular adaptive responses aimed at combating stress and restoring proper protein folding. However, failure to trigger these adaptive changes results in the activation of proteins such as c‐Jun N‐terminal kinase and activating transcription factor 4, exacerbating protein damage and ultimately leading to ER stress and the development of diseases. Furthermore, ER stress can impact the energy metabolism and autophagy process of myocardial cells. In the pathogenesis of DCM, metabolic disturbances, insulin resistance, and various other factors contribute to an imbalance in energy metabolism and a reduction in autophagy levels within myocardial cells. The presence of ER stress compounds these effects by influencing the expression of genes related to energy metabolism and autophagy, thereby exacerbating myocardial cell damage [20].

Calcium signaling exerts a significant regulatory influence on the metabolic processes of myocardial cells. Within the context of DCM, calcium ions are involved in the modulation of numerous pivotal enzymes, consequently impacting the energy metabolism of myocardial cells. Calcium ions possess the ability to stimulate kinases like CaMKII, facilitate the expression and activation of apoptosis‐related molecules, thereby intensifying the apoptotic processes in myocardial cells. Furthermore, calcium ions have the potential to influence mitochondrial functionality and the production of ROS, thereby exerting additional effects on myocardial cell apoptosis [20].

#### 1.1.6 Immune Property Modulation

In DCM, the secretion of cytokines, chemokines, and exosomes by inflammatory cells is implicated in the pathogenesis of cardiomyocyte hypertrophy and extracellular matrix remodeling [3]. Cytokines such as TNF‐α, IL‐6, IL‐1β, interferon‐γ, and TGFβ are known to be involved in cardiac injury [32]. The TLR4 and NLRP3 pathways, as well as 12‐LOX and 15‐LOX, are all directly and indirectly implicated in the development of DCM and HF [3].

#### 1.1.7 Progression of Pathogenic Stages of DCM

**FIGURE 1:**
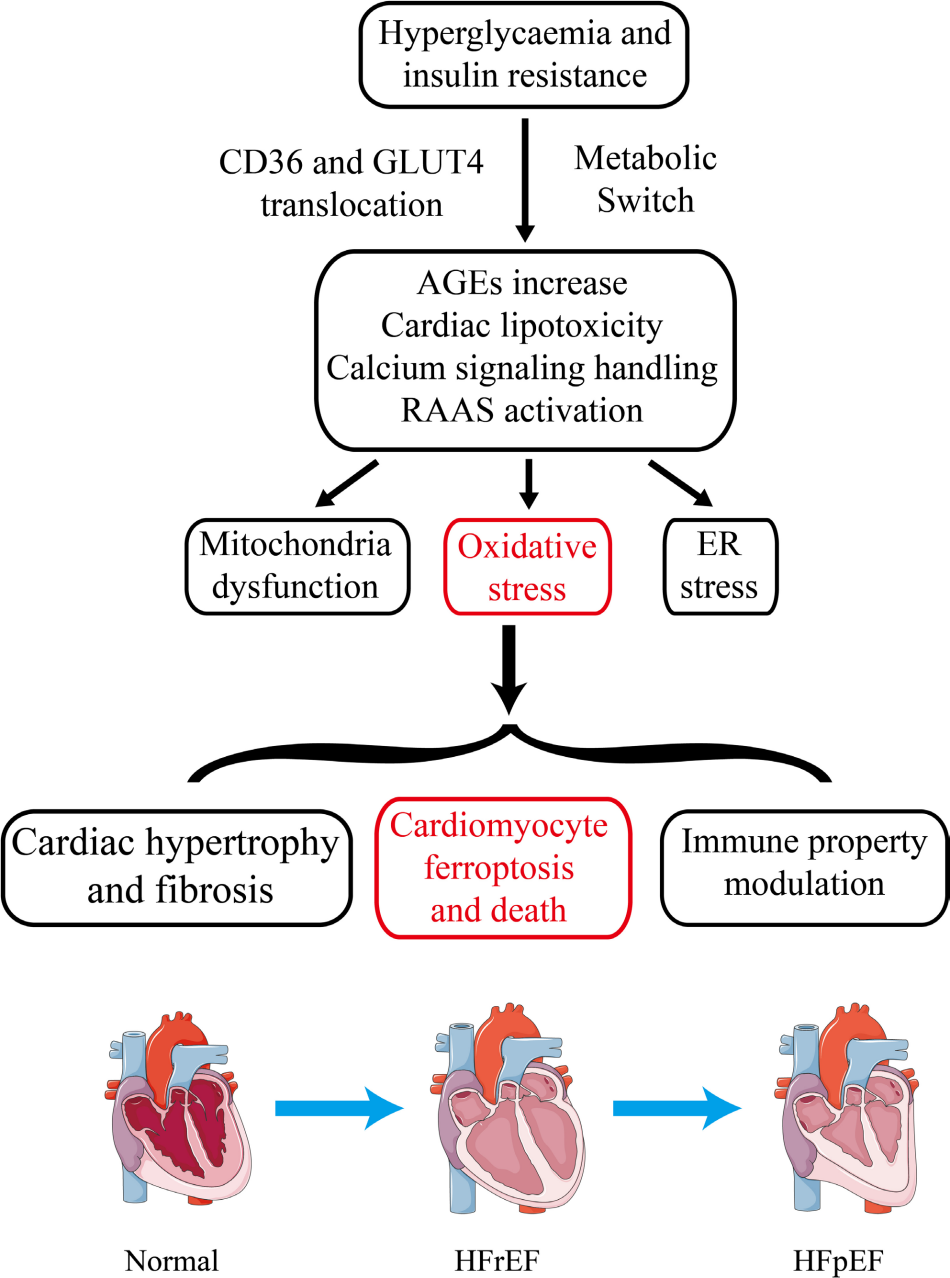
The pathogenesis of DCM.

Early stages of the disease may not exhibit significant changes in myocardial structure or systolic function as assessed by echocardiographic evaluations. The pathogenesis of DCM involves a complex interplay of factors, including CD36 and GLUT4 translocation, insulin resistance, hyperglycemia, altered lipid metabolism, accumulation of AGEs, collagen deposition, and changes in myocardial cell metabolism. These factors contribute to the development of left ventricular stiffness, HF, reduced ejection fraction, and ultimately, diastolic and systolic dysfunction in the late stages of the disease [33]. This process is illustrated in Figure 1.

### 1.2 Clinical Treatment of DCM

Several anti‐HF medications demonstrate comparable efficacy in patients with diabetes and those without diabetes. Conversely, conventional hypoglycemic agents may offer limited cardiovascular protection [10]. Multiple novel diabetes medications demonstrate a cardioprotective effect on the myocardium.

#### 1.2.1 Glucagon‐Like Peptide‐1 (GLP‐1) Analogues

DCM is a prevalent complication of diabetes characterized by myocardial dysfunction and structural changes. Recent research has highlighted the significant therapeutic potential of GLP‐1 in managing DCM [34]. GLP‐1, a hormone secreted by intestinal L cells, exerts various physiological effects. The development of GLP‐1 analogues, including Liraglutide and Lixisenatide, has provided promising alternatives with prolonged half‐lives and enhanced stability compared to native GLP‐1. In the context of managing diabetes‐related cardiomyopathy, these analogues may serve as viable alternatives or adjuncts to GLP‐1 in enhancing insulin sensitivity and cardiac function [35].

#### 1.2.2 Sodium‐Glucose Cotransporter(SGLT) Inhibitors

Recently, sodium‐glucose cotransporter‐2 (SGLT‐2) inhibitors have garnered attention for their potential impact on reducing cardiovascular mortality. Notably, four cardiovascular randomized controlled trials, EMPA‐REG, CANVAS, LEADER, and SUSTAIN‐6, have demonstrated a decrease in cardiovascular events among T2DM patients treated with Empagliflozin, Canagliflozin, liraglutide, and semaglutide, respectively [34]. Based on this data, the U.S. Food and Drug Administration has recently granted approval for Empagliflozin for the purpose of reducing cardiovascular mortality in patients diagnosed with T2DM, marking it as the initial diabetes medication to receive approval for this specific indication [34].

#### 1.2.3 Dipeptidyl Peptidase‐4 (DPP‐4) Inhibitors

Initial studies have indicated that the DPP‐4 inhibitor (Glliptin) has exhibited favorable effects on the cardiovascular and vascular systems, with preliminary findings from phase 2 to 3 clinical trials indicating a decrease in significant cardiovascular events [36]. Subsequent cardiovascular outcome trials of alogliptin, saxagliptin, and sitagliptin did not demonstrate any disadvantages but also did not show any advantages over placebo in individuals with T2DM and high cardiovascular risk [37].

#### 1.2.4 Other New Targets

ROS and oxidative stress are significant factors in the pathogenesis of DCM. Various antioxidant‐based treatment strategies, such as coenzyme Q10 (CoQ10) targeting the PI3K and MAPK pathways, have shown promise in preclinical research [38].

Recent studies have indicated that recombinant human neuregulin‐1 can enhance cardiac function and reverse cardiac remodeling in DCM rats [39, 40]. Huynh et al. [41] conducted a study in which CoQ10 was administered to diabetic mice, resulting in improved levels of pro‐inflammatory markers and diastolic function. Furthermore, the potential efficacy of microRNA‐based treatment and regulation of oxidative stress is suggested in future research.

Despite the rising incidence of DCM, there is currently no established method for prevention or treatment. The prevention and treatment of clinical DCM necessitate a comprehensive approach encompassing dietary management, physical activity, and pharmacological interventions. Currently, therapeutic drugs for DCM are categorized into two main classes: medications for controlling blood sugar levels and drugs for treating HF. The efficacy of glucose‐controlling medications in managing HF is suboptimal, as evidenced by the heightened risk of HF and cardiovascular mortality among patients with T2DM receiving insulin therapy [42]. Metformin may increase the risk of lactic acid poisoning and should be used with caution in patients with symptoms of HF [43]. Similarly, sulfonylureas have been associated with an increased risk of adverse cardiovascular events [44]. Thiazolidinediones have been associated with fluid retention and an increased risk of congestive HF [45]. Certain GLP‐1 agonists have shown potential in mitigating the risk of HF in diabetic patients [46], while the impact of DPP‐4 inhibitors on HF prognosis remains uncertain [37]. Some SGLT2 inhibitors have demonstrated cardioprotective properties [46]. Overall, the influence of glucose‐controlling medications on HF in patients with DCM appears to be constrained, necessitating additional investigation and evaluation. However, clinical drugs used for HF may not be optimal for managing blood sugar levels. Angiotensin‐converting enzyme inhibitors have been shown to enhance insulin resistance and glucose intolerance by upregulating GLUT4 translocation, but they have also been linked to a higher likelihood of hospitalization due to severe hypoglycemia [47]. Statins, which are used to lower lipid levels, have a propensity to cause hyperglycemia [48]. Non‐vasodilating beta‐blockers could exacerbate glycemic regulation, whereas vasodilating beta‐blockers have been found to enhance blood glucose management [49]. Aldosterone receptor antagonists have been shown to enhance left ventricular function in individuals with dilated cardiomyopathy, improve diastolic function, and mitigate fibrosis in those with hypertensive cardiomyopathy and metabolic syndrome. However, the impact of aldosterone antagonists on diastolic function, cardiac insulin resistance, and inflammation in patients with diabetes‐induced HF remains uncertain [50]. In summary, there is currently no designated pharmaceutical intervention for the prevention and management of DCM, necessitating further clinical investigation and research into the efficacy of sugar control and HF medications in specific patient populations. The imperative development of novel therapeutic agents for DCM remains a pressing concern.

## 2 Introduction of Ferroptosis

Preservation of iron homeostasis is crucial for optimal cardiac function [51]. Accumulating evidence suggests that iron dysregulation is a prevalent characteristic across various cardiovascular disorders [51]. In the last decade, ferroptosis, a form of iron‐dependent programmed cell death, has gained recognition as a significant mechanism implicated in the development and advancement of various cardiovascular diseases such as atherosclerosis, drug‐induced HF, myocardial ischemia–reperfusion injury, sepsis‐induced cardiomyopathy, arrhythmia, and DCM [51]. Consequently, enhancing our comprehension of the regulatory pathways governing iron metabolism and ferroptosis in cardiomyocytes may enhance the management of these diseases.

Several types of regulatory cell death, including apoptosis, necroptosis, pyroptosis, and autophagy, have been linked to the pathogenesis of cardiovascular disease [52]. Recent research has increasingly supported the notion that ferroptosis plays a role in the pathophysiology of various cardiovascular diseases, such as doxorubicin‐induced cardiomyopathy, myocardial ischemia–reperfusion injury, myocardial infarction, and HF, over the past decade [51]. Iron, an indispensable trace element ubiquitous in living organisms, plays a crucial role in various biological processes such as energy metabolism and nucleotide synthesis and repair. Iron deficiency, prevalent in up to 75% of individuals with HF, is the most common malnutrition‐related condition in humans [51]. Conversely, both primary and secondary iron overload have been associated with the development of heart disease due to oxidative damage, although the precise mechanism underlying this phenomenon remains unclear. Research has demonstrated that an excess of iron within cardiomyocytes can lead to the induction of ferroptosis through the buildup of phospholipid hydroperoxides in cellular membranes [53]. Furthermore, the excessive generation of ROS or active nitrogen can also directly trigger ferroptosis in cardiomyocytes by facilitating the oxidation of phospholipids in cell membranes [54]. Notably, oxidative stress has been associated with the pathogenesis of cardiovascular disease [55]. In animal models, the targeting of molecules and metabolic pathways that regulate cellular defenses against oxidative stress, specifically the glutathione‐dependent antioxidant system, has demonstrated efficacy in preventing cardiomyopathy [56]. Emerging evidence indicates that the pathogenesis of various cardiovascular diseases is influenced by ferroptosis. Elevated levels of ferroptosis, mediated by diverse signaling and metabolic pathways, have been implicated in the development of ischemic heart disease, cardiac injury, HF, and cardiomyopathy [57].

### 2.1 Major Knowledge of Ferroptosis

The concept of ferroptosis was initially introduced in 2012 by Professor Brent R. Stockwell and his colleagues [58], including Scott Dixon, as a distinct form of iron‐dependent regulated cell death, separate from apoptosis, unregulated necrosis, and programmed necrosis [58]. Ferroptosis is distinguished by lipid peroxidation and can be discerned in cell culture by the restoration of cell viability through the application of iron‐chelating agents and lipophilic antioxidants. The detection of ferroptosis in human and animal tissue samples can be expedited by identifying iron‐dependent lipid peroxidation markers [59]. Since its identification in 2012, ferroptosis has been acknowledged in numerous biological contexts as a mechanism of tissue injury, serving as an evolutionarily conserved form of cellular demise controlled by inherent repair and protective pathways in the absence of cysteine and the presence of TfR1 and glutamine. The widespread occurrence of ferroptosis in various systems highlights the pervasiveness of this type of cell death.

In 2022, Professor Brent R. Stockwell conducted a thorough examination of the tenth anniversary of ferroptosis, offering a systematic survey of the contemporary understanding of the mechanistic pathways involved in this mode of cellular demise. The analysis underscores the pivotal function of lipid peroxidation in propelling ferroptosis, the importance of intracellular ROS in instigating this phenomenon, and the essential role of iron ions in the initiation of ferroptosis [59]. At present, there are three known ways to eliminate peroxidized phospholipids in cells [59]: (1) GPX4 eliminates peroxidized lipids through the action of glutathione (GSH), which is synthesized from cysteine acquired via either the transsulfur pathway or the SystemXc‐transporter. The SystemXc‐transporter facilitates the exchange of intracellular glutamate for extracellular cystine, thereby playing a crucial role in the regulation of ferroptosis. Comprised of solute carrier family 7 member 11 (SLC7A11) and SLC3A2, the SystemXc‐transporter serves as a pivotal component in this cellular process. (2) Ferroptosis suppressor protein 1 (FSP1), also known as apoptosis inducing factor mitochondria associated 2, plays a role in the biosynthesis of CoQ10, which has been shown to possess inhibitory effects on phospholipid peroxidation. The regulation of FSP1 is mediated by MDM2/MDMX‐PPARα signaling pathways and is independent of p53. (3) GCH1 is involved in the biosynthesis of tetrahydrobiopterin (BH4) and contributes to the generation of reduced CoQ10 [59]. Furthermore, ACSL4 plays a role in facilitating the integration of unsaturated fatty acids into phospholipids and also acts as an inhibitor of ferroptosis, although the specific mechanism remains unclear. A recent study by Stockwell et al. [60] revealed that phospholipids containing two polyunsaturated fatty acyl tails are crucial for the occurrence of ferroptosis.

### 2.2 Similarities and Differences Between Ferroptosis and Oxidative Stress

Ferroptosis and oxidative stress are significant physiological processes in cellular biology, exhibiting both similarities and distinctions. These processes serve as crucial signaling pathways within cells, involving the regulation of various pathways such as iron metabolism, ROS metabolism, amino acid metabolism, and lipid metabolism. Furthermore, both ferroptosis and oxidative stress are intricately linked to cell death, with ferroptosis being a regulated form of cell death and oxidative stress also playing a role in cellular death [59]. In some cases, oxidative stress can induce cell apoptosis or necrosis [61]. Major differences between ferroptosis and oxidative stress lie in their regulatory factors. Ferroptosis primarily results from the disruption of key cellular metabolic pathways, such as iron, ROS, amino acid, and lipid metabolism. In contrast, oxidative stress mainly pertains to the balance between ROS production and clearance [62, 63]. The mechanism of ferroptosis differs in its impact on cell survival and death, primarily through the regulation of iron ion and ROS concentrations within cells [59]. The mechanism of oxidative stress primarily involves the overproduction of ROS, leading to cellular damage [62, 63]. The extent of cellular damage is contingent upon the regulatory mechanisms of ferroptosis, a form of programmed cell death that governs cell viability through the modulation of intracellular iron ion concentrations and ROS levels. Oxidative stress, characterized by the overproduction of ROS, contributes to cellular injury [63]. Both ferroptosis and oxidative stress are crucial cellular physiological processes, sharing similarities and differences. They can sometimes promote or inhibit each other, collectively maintaining normal cellular functions.

## 3 Effect of Ferroptosis in the Pathogenesis of DCM

**FIGURE 2:**
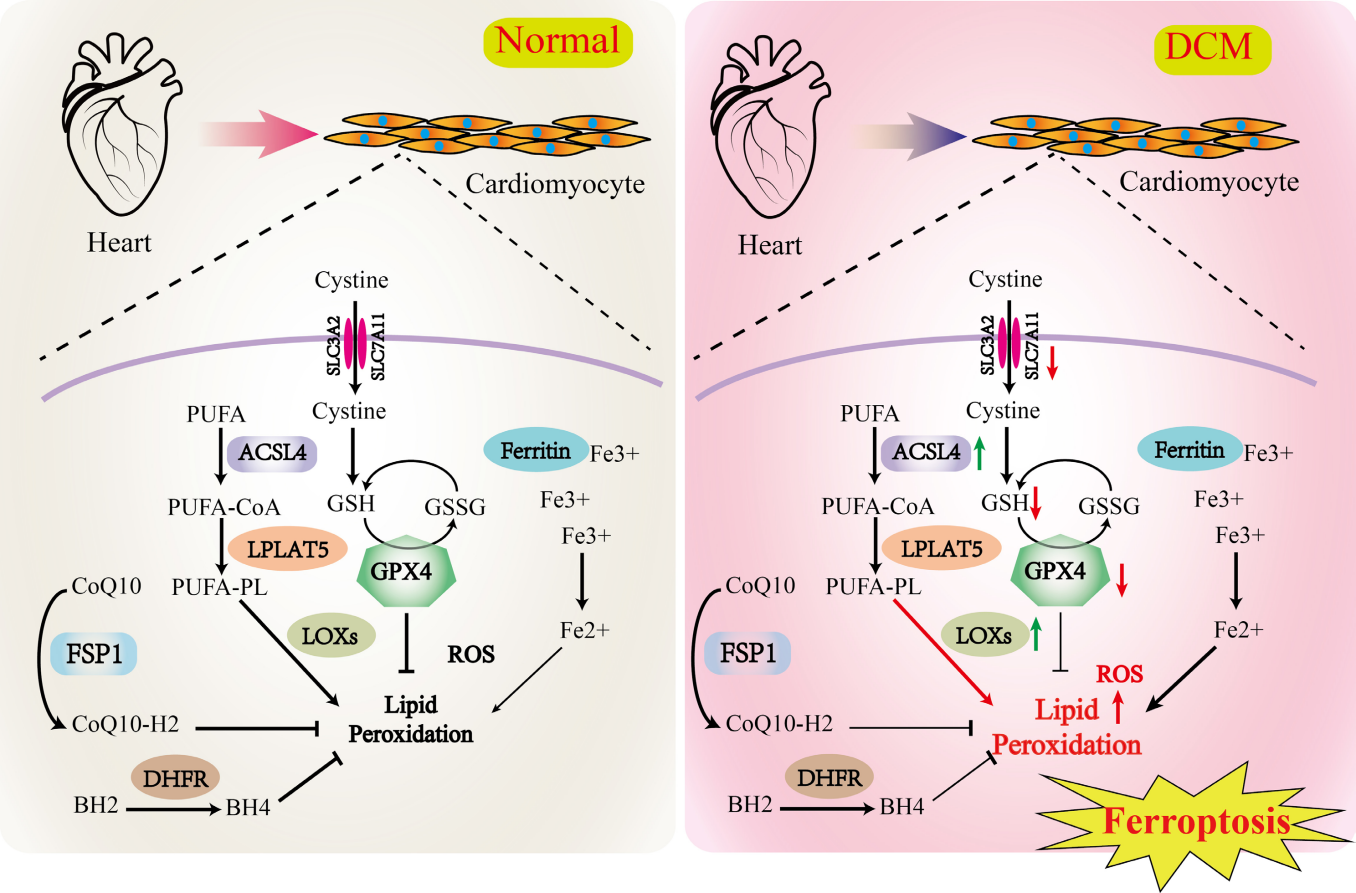
Mechanisms of Ferroptosis in DCM.

Retrospective of the research history of the pathogenesis of DCM, combined with the analysis of ferroptosis‐related phenotypes and the effects of some small molecule compounds, it is clear that ferroptosis plays an important role in the occurrence and development of DCM. Alternatively, research has also found that the use of ferroptosis inhibitors may influence the development and progression of DCM, but more studies are needed to provide evidence. Both iron overload and ROS increasing contribute to the development of DCM. Increasing research reports that iron overload can increase the risk of insulin resistance and contribute to cardiovascular diseases [64, 65]. Oxidative stress is prevalent in DCM and serves as a crucial pathogenic mechanism [20, 62]. Phenotypes, including increased ROS and inactivation of ROS clearance mechanisms, were observed in cardiac tissues. Wu et al. discovered that in a high‐fat diet and STZ‐induced mouse model of DCM, ubiquitin‐specific protease 24 upregulates NF‐κB to deteriorate ferroptosis [66]. Clinical studies have shown significantly elevated ROS levels in the heart muscle and blood vessels of DCM patients. Wang et al. reported that NRF2 activation prevents DCM ferroptosis primarily by increasing ferritin and SLC7A11 expression levels [67]. On the other hand, some ferroptosis inhibitors have been reported to have good efficacy in the prevention and treatment of DCM, which also supports the important role of ferroptosis in the pathogenesis of DCM. In diabetic animal models, certain antioxidants (such as SOD mimics or CoQ10) significantly reverse or delay the disease process, underscoring the role of oxidative stress in the pathogenesis of DCM [68]. Li et al. reported that hypoxia‐reoxygenation (H/R) injury in cultured H9C2 cells and streptozotocin (STZ)‐induced T1DM rat heart tissues can induce ferroptosis and ER stress in cardiomyocytes. The ferroptosis inhibitor Ferrostatin‐1 can mitigate H/R injury [69]. [66]Wang et al. found that palmitic acid (PA) induced ferroptosis in H9C2 through decreased expression of GPX4, while overexpression of heat shock factor 1 alleviates PA‐induced ferroptosis [70]. Wu et al. reported that retinol metabolism is disrupted in T2DM mice and patients. A reduction in retinol dehydrogenase‐10 in cardiomyocytes altered retinol metabolism, inducing DCM through lipotoxicity and ferroptosis [71]. Chen et al. reported that nicorandil alleviates cardiac microvascular ferroptosis in DCM through the mitochondria‐localized AMPK and Parkin‐ACSL4 pathway [72]. However, the intervention with antioxidants like vitamin E has shown less obvious effects, and the specific reasons remain unclear [73]. [67]Additionally, some confirmed ferroptosis inhibitors, such as liproxstatin‐1, an inhibitor of lipid peroxidation, exhibit protective effects on DCM animal models [67]. Sulforaphane can relieve DCM symptoms by activating NRF2 [74]. Resveratrol, a polyphenol antioxidant compound, has been shown to possess inhibitory effects on lipid peroxidation and ferroptosis. Additionally, resveratrol demonstrates a notable ameliorative impact on animal models of DCM [75]. In summary, ferroptosis exacerbates the progression of DCM through mechanisms involving iron accumulation, lipid peroxidation, GPX4 inactivation, inflammatory and immune responses. Understanding the role of ferroptosis in DCM may provide new insights into the pathogenesis of the disease and potential therapeutic targets for its treatment. However, it should be noted that the exact mechanisms and specific contributions by which ferroptosis contributes to DCM progression are still under investigation, and more research is needed to fully elucidate its role and potential as a therapeutic target. The involvement of ferroptosis in DCM is depicted in Figure 2.

## 4 Potential Value of Ferroptosis Inhibitors in DCM Intervention

Targeting ferroptosis may represent a promising therapeutic approach for DCM. Wang et al. have demonstrated that ferroptosis plays a crucial role in the pathogenesis of DCM, and that the ferroptosis inhibitor liproxstatin‐1 has the potential to mitigate diastolic dysfunction in diabetic individuals [67]. Sulforaphane, a newly discovered reagent that inhibits ferroptosis, can also defend against DCM by increasing the expression of SLC7A11 [67]. The SGLT2 inhibitor canagliflozin may exert some of its cardiovascular benefits by reducing ferroptosis [76]. Canagliflozin mitigates lipotoxicity in cardiomyocytes by inhibiting inflammation and ferroptosis through activation of the AMPK pathway [77]. Li et al. [78] reported that dexmedetomidine can prevent DCM by inhibiting ferroptosis. Nicorandil, Astragaloside IV, and Curcumin demonstrate potential therapeutic effects in alleviating cardiac microvascular ferroptosis in DCM through various signaling pathways, including the mitochondria‐localized AMPK‐Parkin‐ACSL4 pathway, CD36‐mediated ferroptosis downregulation, and the Nrf2 pathway, respectively [72, 79, 80]. Suberosin demonstrates efficacy in mitigating thiazolidinedione‐induced cardiomyopathy in diabetic rats through the inhibition of ferroptosis, achieved by the modulation of ACSL4‐LPCAT3 and PI3K‐AKT signaling pathways [81]. Paeoniflorin confers resistance to ferroptosis by modulating the gut microbiota and its metabolites in DCM [82]. Additionally, 6‐Gingerol alleviates ferroptosis and inflammation in DCM through activation of the Nrf2/HO‐1 pathway [83]. Generally, several ferroptosis inhibitors hold great promise as therapeutic agents for the intervention of DCM. Their ability to reduce iron‐mediated oxidative stress, inhibit expression and function of GPX4, preserve mitochondrial function, attenuate inflammatory responses, modulate cellular signaling pathways, and potentially synergize with anti‐diabetic medications makes them valuable candidates for further research and development. However, the exact mechanisms of action and therapeutic efficacy of these inhibitors in DCM remain to be fully elucidated through clinical trials and further research. The ferroptosis inhibitors that have been reported in the literature to be effective against DCM are summarized in Table 1.

**TABLE 1:** Emerging compounds targeting ferroptosis to inhibit DCM.

Compounds	Models	Mechanism and effect	Refs.
Sulforaphane	Rat/HFD/STZ	↓ACSL4; ↑ Nrf2 and FPN1; ↑GPX4	[84]
	C57 mice/HFD/STZ	↑Ferritin; ↑SLC7A11; ↑ GSH; ↓Fe^2+^; ↓MDA	[81, 85]
	H9C2/high glucose	↓ACSL4; ↑Nrf2 and FPN1; ↑GPX4	[81, 86]
6‐Gingerol	C57 mice/HFD/STZ	↓FACL4; ↓Fe^2+^; ↑GPX4; ↑Nrf2 pathway; ↑SOD; ↓MDA	[87]
	H9C2/high glucose	↓FACL4; ↓Fe^2+^;↑GPX4; ↑Nrf2; ↑SOD; ↓MDA	[88]
Curcumin	Rabbit/STZ	↓ACSL4 and COX2; ↑Nrf2; ↑GPX4	[80, 89]
	H9C2/high glucose	↑Nrf2, GPX4; ↓ROS	[90]
Canagliflozin	C57 mice/STZ	↓MDA; ↑SOD; ↑CAT; ↑GSH; ↓deposition of total iron and Fe^2+^; ↓FTH; ↑SLC7A11	[76]
	HL‐1/PA	↓ROS; ↓MDA; ↑GSH; ↓deposition of total iron and Fe^2+^	[77]
	H9C2/high glucose	↓ROS; ↓Lipid‐ROS; ↓MDA; ↑SOD;↑GSH; ↓deposition of total iron and Fe^2+^; ↓FTH; ↑SLC7A11	[76]
SR9009	Rat/HFD/STZ	↓ferritinophagy/ferroptosis‐related proteins	[91]
Tadalafil	db/db mouse model of T2DM	↓ROS; ↓lipid peroxidation	[92]
Liproxstatin 1	C57 mice/HFD/STZ	↑SLC7A11; ↑ Ferritin; ↑GSH;↓MDA	[67]
N‐Acetyl cysteine	Rat/STZ	↓MDA; ↑Gpx4; ↑Nrf2	[93]
Dexmedetomidine	H9C2/high glucose	↓MDA; ↓ROS; ↑SOD2; ↑GPX4	[78]
Resveratrol	C57 mice/HFD/STZ	↓oxidative stress	[94]
Curcumin	Rabbit/STZ	↑GPX4; ↓ACSL4; ↑Nrf2	[80]
	Rat/STZ	↑Nrf2; ↓ROS; ↑SOD	[88]
Spermine	Rat/STZ	↓ROS	[95]
Ferrostatin‐1	AGEs Treated engineered cardiac tissues	↓MDA; ↓Iron overload	[67]
Deferoxamine	AGEs Treated engineered cardiac tissues	↓MDA;↓Iron overload	[67]
Vitamin E	Rat/STZ	↓Oxidative stress	[96]
Broccoli sprout extract	db/db mouse model of T2DM	↓MDA;↓Oxidative stress	[97]
mito‐TEMPO	Mice/STZ and db/db mouse model of T2DM	↓ mitochondrial ROS;↓oxidative stress	[98]
Astragalus polysaccharide	H9C2/high glucose	↓ROS; ↑SOD	[99]
Allopurinol	Rat/STZ	↓ROS	[100]
	H9C2/high glucose	↓ROS	[100]
Luteolin	Mice/STZ	↓oxidative stress; ↓MDA	[101]
	Rat/STZ	↓MDA; ↑SOD; ↑GPX; ↑Catalase	[101]
	H9C2/high glucose	↓oxidative stress	[102]
L6H21	Mice/HFD	↓oxidative stress; ↓ ACSL4; ↑ GPX4	[103]
Nicorandil	db/db mouse model of T2DM	↓ ACSL4; ↑ GPX4;↓deposition of total iron and Fe^2+^	[72]
Astragaloside IV	Rat/STZ	↑GPX4; ↓ACSL4; ↓CD36	[79]
Paeoniflorin	Mice/STZ	↑GPX4; ↓MDA; ↓deposition of total iron and Fe^2+^; ↑GSH	[82]
Suberosin	Rat/STZ	↓LOX; ↓ACSL4; ↓LPCAT3; ↑GPX4	[81]
Empagliflozin	KK‐Ay mice (genetic T2DM model)	↓MDA; ↓ROS; ↑SOD	[104]

## 5 Conclusions

The involvement of ferroptosis in cardiomyocytes is significant in the pathogenesis of DCM, as it encompasses various metabolic pathways, including iron, lipid, and glutathione metabolism. Investigation into ferroptosis offers novel avenues for therapeutic drug development for DCM, with specific inhibitors potentially serving as promising treatments. These inhibitors have the potential to modulate key processes such as iron metabolism, lipid peroxidation, and the glutathione system, ultimately impeding the onset of ferroptosis. These potential drugs may also be considered for combination therapy with other cardiovascular disease medications. For instance, certain hypoglycemic agents, antiplatelet medications, or treatments for cardiovascular disease may exhibit inhibitory effects on ferroptosis. Consequently, the combination of these medications could potentially enhance the efficacy of treatment for DCM.

In summary, the potential therapeutic significance of ferroptosis in the management of DCM is substantial. By investigating targeted therapy, drug development, and combination therapy, novel treatment approaches may be elucidated, offering innovative perspectives and methodologies for addressing DCM. Nonetheless, current research on ferroptosis in DCM remains incomplete, necessitating further investigation into its underlying mechanisms and clinical efficacy for future advancements.

## Author Contributions

All authors contributed to writing the manuscript and read and approved its final version.

## Ethics Statement

The authors have nothing to report.

## Consent

The authors have nothing to report.

## Conflicts of Interest

The authors declare no conflicts of interest.

## Data Availability

The authors have nothing to report.
